# Effectiveness of acupuncture for multiple sclerosis: A protocol for systematic review and meta-analysis

**DOI:** 10.1097/MD.0000000000029150

**Published:** 2022-04-01

**Authors:** Hong Guan, Jingyu Wang, Yongzheng Zhu, Hongling Jia, Yongchen Zhang

**Affiliations:** a Acupuncture and Tuina College, Shandong University of Traditional Chinese Medicine, Shandong, China; b Acupuncture & Moxibustion Department, The Second Affiliated Hospital of Shandong University of Traditional Chinese Medicine, Shandong, China; c Acupuncture & Moxibustion Department, Affiliated Hospital of Shandong University of Traditional Chinese Medicine, Shandong, China.

**Keywords:** acupuncture, meta-analysis, multiple sclerosis, protocol

## Abstract

**Background:**

Multiple sclerosis (MS) is an autoimmune disease characterized by white matter inflammatory demyelination in the central nervous system (CNS). Its clinical manifestations include decreased vision, diplopia, and limb weakness. As a green and simple traditional Chinese medicine method, acupuncture is gradually recognized by the public. At present, there is still a lack of systematic evaluation on acupuncture treatment of MS. This study aims to evaluate the safety and effectiveness of acupuncture in the treatment of MS, in order to provide a basis for clinical decision-making.

**Methods:**

Randomized controlled trials (RCTs) of acupuncture for MS will be searched in the relevant database, including PubMed, MEDLINE, Web of Science, Embase, Cochrane Library, China National Knowledge Infrastructure Database (CNKI), WanFang Database, China Biology Medicine Database (CBM), Chinese Scientific Journals Database (VIP), regardless of publication date, or language. All relevant RCTs of electronic searches will be exported to EndNote X9.1 software. Data analysis will be performed using RevMan 5.4 and STATA 14.2 software.

**Results:**

Our study aims to explore the efficacy of acupuncture for MS and to provide up-to-date evidence for clinical of MS. We will publish our research results in peer review journals.

**Conclusion:**

This study will perform a comprehensive systematic review and meta-analysis on the efficacy of acupuncture for MS, making up for the lack of relevant evidence of the clinical use of acupuncture.

## 1 Introduction

Multiple sclerosis (MS) is a chronic, immune-mediated central nervous system (CNS) disease. Its pathological features are inflammation, demyelination, and axonal injury. It has the characteristics of multiple spatial and temporal, recurrent attacks, and high disability rate. According to epidemiological statistics, the prevalence and incidence of MS varies from 2/100,000 in Japan to 100/100,000 in North Europe and North America.^[1]^ At present, its pathogenesis is not fully understood and may be related to genetic, environmental, and viral factors.^[2]^ The treatment of this disease in modern medicine mainly adopts hormone, immunosuppressant, plasma exchange, and other therapies, with common adverse reactions such as diarrhea and vomiting.^[3]^ As a clinically intractable disease, MS has caused a large medical burden to the patients themselves and the current society.^[4]^ Therefore, how to treat MS safely and effectively has become an urgent social medical problem.

As a famous complementary and alternative medicine method, acupuncture has been widely used in the treatment of stroke,^[5]^ Parkinson,^[6]^ spinal cord injury,^[7]^ and other neurological diseases, may become a potential choice for the treatment of MS. Current studies have shown that acupuncture as a single or adjuvant therapy has a positive effect on symptom relief in patients with MS.^[8]^ Although there are some original literatures on acupuncture treatment of MS, the relevant meta-analysis is still relatively blank. Therefore, in order to prove whether acupuncture is really effective in the treatment of MS, we will use all relevant randomized controlled trials (RCTs) data for meta-analysis to comprehensively evaluate the effectiveness and safety of acupuncture in the treatment of MS, and provide a basis for clinical practice.

## 2 Methods and analysis

### 2.1 Study registration

The study has been registered on the International Platform of Registered Systematic Review and MetaAnalysis Protocols (INPLASY). The approved registration number is INPLASY202220121 (DOI:10.37766/inplasy2022.2.0121). And it was built on the guidelines of PRISMA-P (Preferred Reporting Items for Systematic Reviews and Meta-Analysis Protocols).^[9]^

### 2.2 Inclusion criteria

#### 2.2.1 Types of studies

RCTs of acupuncture in the treatment of MS were comprehensively searched, regardless of language or publication date. In addition, manually search for unpublished documents. The literature included should be original articles of peer review. Excluding reviews, case reports, animal trials, mechanism studies, and other unpublished studies.

#### 2.2.2 Types of participants

The included studies should be those who have been clearly diagnosed with MS. Age between 18 and 65 years old, no restrictions on gender, education, race, or disease stage.

#### 2.2.3 Types of interventions

The experimental group is defined as acupuncture treatment, such as manual acupuncture, warm needling moxibustion, electroacupuncture, auricular acupuncture, fire needling, elongated needle, or moxibustion.

#### 2.2.4 Comparison group type

The control group that will include non-acupuncture techniques, such as sham acupuncture, placebo, adjuvant chemotherapy, or other pharmacotherapy. The acupoint numbers, retaining time, and frequency will not be restricted in this protocol.

#### 2.2.5 Types of outcome measures

##### 2.2.5.1 Primary outcomes

The main outcome indicators included expanded disability status scale (EDSS),^[10]^ total effective rate, clinical symptom score, and neurological function score.

##### 2.2.5.2 Secondary outcomes

Secondary outcome indicators include the number of annual recurrences, adverse reactions.

### 2.3 Exclusion criteria

Studies that are repeatedly published and necessary information cannot be obtained in various ways will be excluded.

### 2.4 Database search strategy

#### 2.4.1 Electronic researches

The electronic databases of PubMed, MEDLINE, Web of Science, Embase, Cochrane Library, China National Knowledge Infrastructure Database (CNKI), WanFang Database, China Biology Medicine Database (CBM), and Chinese Scientific Journals Database (VIP) will be retrieved. The time span of electronic searching is from the start date to February 2022. We will also manually search unpublished studies and references. The search strategy that will be run in the PubMed and adjusted to fit the other database when necessary is presented in Table 1.

**Table 1:** PubMed search strategy.

Number	Search items
#1	(“Multiple Sclerosis”[Mesh]) OR (((((Sclerosis, Multiple[Title/Abstract]) OR (Sclerosis, Disseminated[Title/Abstract])) OR (Disseminated Sclerosis[Title/Abstract])) OR (MS (Multiple Sclerosis[Title/Abstract]))) OR (Multiple Sclerosis, Acute Fulminating[Title/Abstract]))
#2	“Acupuncture” [Title/Abstract] OR “Moxibustion” [Title/Abstract] OR “Electroacupuncture” [Title/Abstract] OR “Fire needle” [Title/Abstract] OR “Auricular point” [Title/Abstract] OR “Warming needle moxibustion” [Title/Abstract]
#3	“Randomized controlled trial” [Title/Abstract] OR “Controlled clinical trial” [Title/Abstract] OR “Clinical trial randomized” [Title/Abstract]
#4	#1 and #2 and #3

#### 2.4.2 Search for additional resources

We will search for ongoing or unpublished studies from ClinicalTrials.gov, China Clinical Trials Registry, PROSPERO, National Institutes of Health Registry, and World Health Organization International Clinical Trials Registry.

### 2.5 Selection of studies

**Figure 1:**
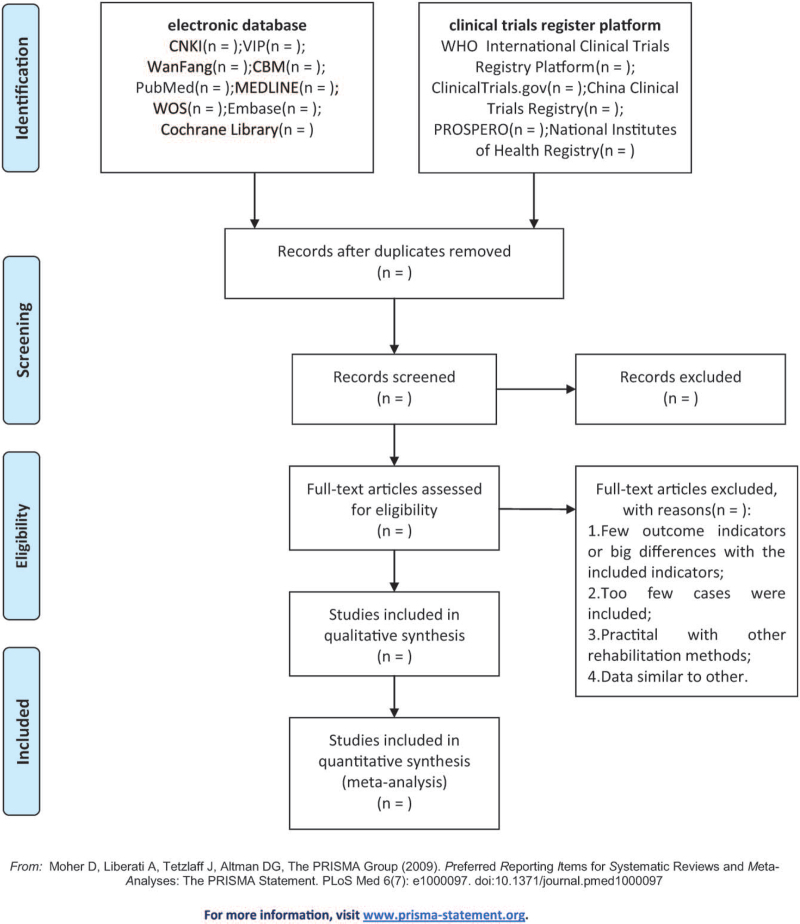
The PRISIMA flow diagram.

All the retrieved literature research will be imported into EndnoteX9.1 for management, and the repeated literature will be deleted. Two researchers (HG and JYW) will independently undertake the search results selection process according to the inclusion and exclusion criteria. They will review and screen through titles and abstracts retrieved from the literature to exclude unrelated research trials. Any problems in the literature screening will be resolved by two authors. For inconsistent studies, let a third researcher (YZZ) decide. The selection process is shown in the PRISMA flowchart (Fig. 1).

### 2.6 Data extraction and management

The data will be independently extracted from the selected articles by two researchers (HG and JYW) using Excel spreadsheet. The extracted content included the first author, publication time, research type, sample size, characteristics of participants, intervention measures, treatment process, outcome indicators, and adverse events in the treatment group and the control group. Differences in data extraction are resolved through discussions with a third (YZZ) researcher.

### 2.7 Dealing with missing data

We will attempt to contact authors to obtain missing data. If we cannot contact the original authors, the studies will be excluded from the data synthesis.

### 2.8 Methodology quality assessment

Two researchers (HG and JYW) will use the “bias risk assessment tool” recommended in the Cochran Manual 5.1.0 to assess the methodological quality of RCTs.^[11]^ It includes seven items: random sequence generation, allocation concealment, blindness of participants and caregivers, blindness of outcome evaluators, incomplete outcome data, selective outcome reports and other biases. The evaluation results of each project will be divided into high risk, low risk or unclear risk. The assessment will be completed by two researchers and dissent will be submitted to a third researcher (YZZ) for final decision.

### 2.9 Assessment of heterogeneity

According to the heterogeneity test, Chi-square test and I^2^ statistics should be used to evaluate the statistical heterogeneity, and the use of fixed effect model or random effect model should be determined according to the results of I^2^ statistics. When *P* > .05 or I^2^ < 50%, choose fixed effect model. When *P* < .05 or I^2^ ≥ 50%, random effect model was selected. When the results are not suitable for merger, we will conduct descriptive analysis. When more than 10 studies are included, we will use funnel plot to evaluate publication bias. The symmetry of funnel plot means no publication bias.^[12]^

### 2.10 Statistical analysis

This study will use RevMan 5.4 (Cochrane Collaboration, Nordic Cochrane Center, Copenhagen, Denmark) software and Stata 14.2 (Stata Corp, College Station, TX) for data analysis and synthesis. Select different measurement indexes according to different data types. In the analysis of continuous results, the mean difference (MD) or standard mean difference (SMD) of 95% confidence interval (CI) is used as a statistical indicator, and the relative risk (RR) or ratio (OR) of 95% CI is used to analyze and evaluate the therapeutic effect of dichotomy results.

### 2.11 Subgroup analysis

If there is significant heterogeneity among the research results, we will conduct subgroup analysis to explore the reasons for heterogeneity.

### 2.12 Sensitivity analysis

We will conduct sensitivity analysis to verify the robustness of the results. It includes methodological quality, research design, and impact of sample size.^[13]^

### 2.13 Quality of evidence

The quality of evidence will be evaluated by the Grading of Recommendations Assessment, Development and Evaluation (GRADE) method.^[14]^ There are four levels: very low, low, medium, or high.

## 3 Discussion

MS is an autoimmune disease characterized by demyelination of CNS and continuous inflammatory response, with high morbidity and mortality. Its pathogenesis is complex, and some of the pathological mechanisms are not yet fully understood. At present, it has been found that immune disorders are caused by genetic and environmental factors.^[15]^ Acupuncture is a kind of green therapy with less pain, which can modulate the plasticity of CNS, effectively improve the transmission of nerve information, regulate the endocrine system, and play an active role in the treatment of MS.^[16–18]^ However, its efficacy has not been scientifically and systematically evaluated. To address this limitation, this study will collect large sample, multicenter, high-quality RCTs to test the efficacy and safety of acupuncture in the treatment of MS. This review also has some limitations. Different acupoint selection methods and disease severity may lead to heterogeneity.

## Author contributions

**Methodology:** Jingyu Wang, Yongzheng Zhu.

**Resources:** Jingyu Wang, Yongzheng Zhu.

**Software:** Jingyu Wang, Hongling Jia.

**Supervision:** Hongling Jia.

**Validation:** Yongzheng Zhu.

**Visualization:** Yongchen Zhang.

**Writing – original draft:** Hong Guan.

**Writing – review & editing:** Yongchen Zhang.
